# Internet-based modular program BADI for adjustment disorder: protocol of a randomized controlled trial

**DOI:** 10.1186/s12888-016-0980-9

**Published:** 2016-07-26

**Authors:** Paulius Skruibis, Jonas Eimontas, Migle Dovydaitiene, Egle Mazulyte, Paulina Zelviene, Evaldas Kazlauskas

**Affiliations:** 1 Department of Clinical and Organizational Psychology, Vilnius University, Universiteto str. 9/1, Vilnius, 01513 Lithuania; 2 Department of General Psychology, Vilnius University, Universiteto str. 9/1, Vilnius, 01513 Lithuania

**Keywords:** Adjustment disorder, Internet-based, Online, intervention, EHealth, RCT, Stress

## Abstract

**Background:**

Adjustment disorder is one of the most common mental health diagnoses. Still it receives relatively little attention from researchers trying to establish best interventions to treat it. With high prevalence of stressful life events, which might be leading to adjustment disorder, and limited resources of mental health service providers, online interventions could be a very practical way of helping people who have these disorders or are in the risk to develop them. The proposed study protocol is aimed to describe a randomized controlled trial of an internet-based modular intervention for adjustment disorder as it is defined in a proposal for the ICD-11.

**Methods/design:**

This study is a two-armed Randomized Controlled Trial (RCT) to examine the effectiveness of a web-based intervention BADI (Brief Adjustment Disorder Intervention) for adjustment disorder symptoms. BADI has four modules: Relaxation, Time management, Mindfulness and Strengthening relationships. It is based on stress and coping research and integrates evidence-based treatment approaches such as Cognitive Behavioural therapy (CBT), mindfulness and body-mind practices, as well as exercises for enhancing social support. Primary outcome of the study are symptoms of adjustment disorder and well-being. Engagement into the program and motivation for change is a secondary outcome. All participants after completing the baseline assessment are randomly assigned to one of the two groups: either to the one in which participant will instantly gain access to the BADI intervention or a group in which participants will be given access to the BADI program after waiting one month. Participants of BADI can choose exercises of the program flexibly. There is no particular order in which the exercises should be completed.

**Discussion:**

Study will provide new insights of modular internet-based interventions efficacy for adjustment disorders. The study will also provide information about the role of motivation and expectancies on engagement in modular internet-based interventions. In case this RCT supports effectiveness of fully automated version of BADI, it could be used very broadly. It could become a cost-effective and accessible intervention for adjustment disorder.

**Trial registration:**

The study was retrospectively registered with the Australian and New Zealand Clinical Trials Registry with the registration number ACTRN12616000883415. Registered 5 July, 2016.

## Background

### The new concept of adjustment disorder

Adjustment disorder is one of the most common mental health diagnoses around the world [1]. Despite that, it has been considered a poorly defined area of psychopathology, therefore conceptual changes were proposed both for DSM-5 and ICD-11 [2–5].

In DSM-5 adjustment disorders have been classified under trauma and stress related disorders for the first time. However, some scholars argue that they still remain rather loosely defined [5]. Proposals for ICD-11 conceptualize adjustment disorder as a maladaptive reaction to identifiable stressors [4]. Intrusive preoccupations with the stressor, avoidance and failure to adapt should separate this disorder from normal reactions and no requirements for severity of the stressor should distinguish it from PTSD [3, 4, 6].

This new conception of adjustment disorder, that was proposed for ICD-11, was employed in a representative national wide survey of general population in Germany [2]. Results of the study indicated 0.9 % prevalence of adjustment disorder fulfilling clinically significant impairment criterion, and 1.4 % without fulfilling this criterion. This study also established life events that were most frequently associated with adjustment disorder: serious illness, conflict with neighbours and job-related conflict. The proposed new structure of the adjustment disorder was also recently validated in a Lithuanian representative population sample [7]. This study supported the two core symptom adjustment disorder structure.

### Interventions for Adjustment disorder

In addition to vagueness of definitions, adjustment disorder received relatively little attention from researchers trying to establish best interventions to treat it [5, 8]. As evidence for the benefits of pharmacological treatment is extremely limited, psychotherapeutic interventions are considered as treatment of choice [5, 8, 9]. Among specific psychotherapeutic approaches the following were tried without clear consensus which are the most effective: supportive, psychoeducational, cognitive, psychodynamic, Interpersonal, ego-enhancing, problem-solving, eye movement desensitization and reprocessing, support groups, “mirror psychotherapy” and “activating intervention” [5, 8, 9].

### E-health advantages in treating adjustment disorder

Having in mind high prevalence of stressful life events, which might be leading to adjustment disorder, and limited resources of mental health service providers, online interventions could be a very practical way of helping people who have these disorders or are in the risk of developing them. Especially if these interventions could be provided automatically, thus requiring little human resources.

Studies of another more severe stress related disorder – posttraumatic stress disorder (PTSD) – show that stigmatization of help, practical issues such as incompatible time, transportation problems, financial problems, lack of professional services in residential area, negative prejudice about the effectiveness and availability of help are among those barriers that prevent people from getting help they really need [10, 11]. Internet-based interventions might be a good way to remove at least some of these obstacles for treatment.

A meta-analysis of the effectiveness of internet-based psychotherapeutic interventions for various conditions showed that these interventions could be as effective as face to face therapies [12]. A more recent meta-analysis of telehealth treatments supported the use of telehealth treatments specifically for individuals with PTSD-related symptoms [10]. To our knowledge there are only two telehealth interventions developed specifically for adjustment disorders [8]. Both interventions have not been evaluated in large-scale empirical studies yet.

We have developed a web-based intervention BADI (Brief Adjustment Disorder Intervention) to help people to deal with adjustment disorder symptoms after stressful life events. This paper reports on the protocol of a RCT examining the effectiveness of this intervention.

### Aims of the study

There are two aims of this RCT: 1) to evaluate the effectiveness of an internet-based modular intervention for adjustment disorder (BADI); 2) to examine correlations between effectiveness, motivation, expectations, drop-out and adherence.

## Methods/design

**Fig. 1:**
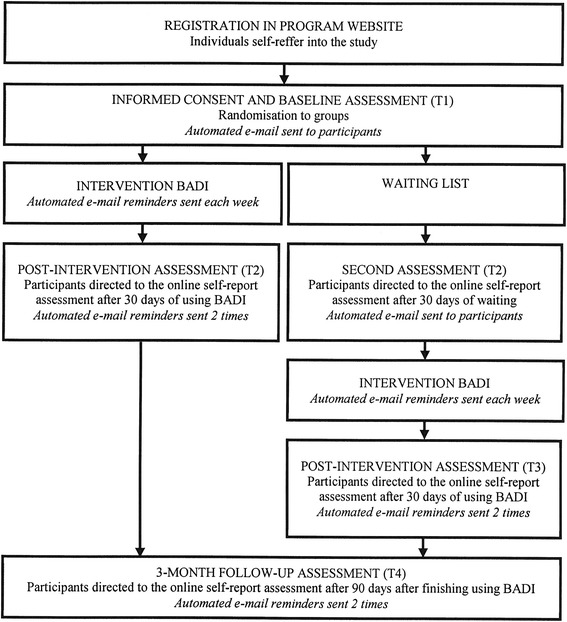
Flow chart of the study

BADI is an open access internet-based modular intervention for adjustment disorders registered with the Australian and New Zealand Clinical Trials Registry with the registration number ACTRN12616000883415. The study was considered and approved by Vilnius University Psychology Research Ethics Committee. This study follows CONSORT E-HEALTH guidelines [13]. Figure 1 represents procedure of the study schematically.

### Setting

Participation in the study is online only. Vilnius University is the administering institution of the study website.

### BADI intervention

BADI program is a structured short-term online intervention program addressed to people with adjustment disorders or those who are experiencing stressful life events and have a risk for adjustment disorders. BADI program is focused upon enhancing process of psychological resilience, utilizing personal and social resources and developing better coping skills.

The program consists of four modules: Relaxation (named “Body”), Time management (named “Time”), Mindfulness (named “Space”) and Strengthening relationships (named “Relationships”). Descriptions of exercises are provided in Table 1.

**Table 1:** BADI content

*Module*	*Exercise*	*Aim of the exercise*	*Activity in the module*
Relaxation “*Body”*	Progressive muscle relaxation	Relieving muscle tension, relaxing body, enhancing stress resistance. Learning how to actively relax in stressful situations	Progressive relaxation using voice-guided instructions. Participant performs progressive muscle relaxation exercise. Duration 5-10 min.
	Breathing awareness	Recognising breathing pattern and relaxing	Structured self-observation exercise. Participant concentrates on breathing and present moment. Duration 5-10 min.
	Body scanning	Focusing mindful attention, reducing tension, achieving mind-body balance	Voice-guided structured exercise. Participant focuses on breathing and sensations. Duration 5-10 min.
Time management “*Time”*	Priorities of the day	Recognising core values, choosing between alternatives, establishing personal guidelines	Voice-guided fantasy exercise and pencil-paper list making. Participant plans and implements important day activities. Duration 5-15 min.
	Accomplishment	Revealing valuable task of the day, planning action and execution	Participant writes down three tasks, then chooses one and divides it into smaller pieces. The participant then makes a schedule for those divided taskas. Duration 5-10 min.
	Planning pleasant activities	Making decisions, taking actions, preventing distress, creating positive experiences	Brainstorming, planning and implementing homework assignment. Participant plans and implements pleasant activity. Duration 5-10 min.
Mindfulness “*Space*”	Walking meditation	Establishing posture, concentrating on action, relaxing and focusing attention	Voice-guided structured exercise. Participant performs relaxation and meditation while walking. Duration 5-10 min.
	Seeing meditation	Anchoring attention, relaxing and concentrating on the moment	Voice-guided structured exercise. Participant performs sight concentration exercise focusing attention at the moment. Duration 5-10 min.
	Letting go of thoughts	Concentrating on sensations, relieving negative thoughts and tension	Voice-guided structured exercise. Participant performs visualisation exercise. Duration 5-10 min.
Strengthening relationships “*Relationships*”	Partners’ encouragement	Developing empathy, sharing partners’ feelings, coping with interpersonal stress and preventing conflict	Reflexion, structured action planning and implementation exercise. Participant is asked to choose one way to encourage partner and realise this at the moment. Duration 10-15 min.
	Assertive message	Sharing expectations, training assertiveness, preventing conflict and increasing social support	Reflexion, structured action planning and implementation exercise. Participant reflects upon important people and shares assertive message to them. Duration 5-15 min.
	Appreciation	Expressing appreciation, encouraging cooperation, strengthening connection	Reflexion, structured action planning and implementation exercise. Participant shares ones needs and appreciation with the partner. Duration 5-10 min.

BADI program is based on stress and coping research and integrates evidence-based modern treatment approaches such as Cognitive Behavioural therapy (CBT), mindfulness and body-mind practices, as well as exercises for enhancing social support.

Relaxation module exercises were selected considering research on the topic. Progressive muscle relaxation effectiveness studies demonstrated that it could be a useful technique in various fields of medicine [14–16]. BADI relaxation module also includes breath and body scanning exercises which are based on combined body and mindfulness principles [17–20].

The focus of Time management module is to stimulate problem-focused stress coping [21]. The analysis of coping strategies revealed that problem-focused coping is more efficient than emotion-focused [22] and is related to greater health benefits and better adjustment.

BADI mindfulness module is based on a review of rapidly growing evidence of effect of mindfulness on stress and well-being (e.g. [23–26]).

Social support is another important part of effective coping with stress [27]. BADI relationships module includes partner encouragement exercise, assertive message and appreciation which enhance stress coping through open communication and social support [18, 21].

Exercises take from 5 to 15 min to complete. The participant may complete as many or as few modules as they choose. This enables a participant to personalize the intervention to his needs. The participants are instructed that they will have access to BADI for one month.

### Participants

#### Inclusion criteria

All individuals willing to participate in the study have to have adjustment difficulties and be no less than of 18 years old, have access to internet and a computer or a smart gadget with screen and audio output, and also have sufficient Lithuanian language literacy to understand the instructions and give informed consent.

#### Exclusion criteria

Risk of suicide, severe adjustment difficulties.

#### Withdrawal criteria

Individuals who wish to terminate their participation in the program are asked if they would consent to completing following assessments. If not, no further remainder e-mails or invitations to complete assessments are sent.

#### Recruitment

All participants are self-referred. Study is advertised via social media and media, and can be accessed via Facebook links, and media articles.

#### Enrolment

Participants interested in the study can register to the program at the intervention website. Eligible participants are then provided with detailed information about the study and are asked to give an informed consent. After giving informed consent and filling in the self-report measures of the initial assessment (T1) they are asked to wait until they are approved for the participation.

#### Randomization

The randomization process is continuous in a way that the participant is instantly allocated to the intervention or waiting-list control group within 24 h of completing the initial assessment. Randomization is conducted by study team member using an online true random number service www.random.org. Participants are randomized in 1:1 ratio to study groups. No stratification is applied.

#### Blinding

Participants are not blinded to possible groups. Participants are informed that after completing the baseline assessment they would be randomly assigned to one of the two groups: either to the one in which participant will instantly gain access to the BADI intervention or a group in which participants will be given access to the BADI program after waiting for one month.

### Study groups

#### Intervention BADI

Participants in the intervention BADI group have full access to the intervention modules for 30 days. Participants can freely select any of the BADI intervention exercises. Participants can complete as many or as little exercises as they need. Participants can do exercises in any order they prefer. There are no instructions to complete all exercises. Participants can choose to complete one or more exercises and do them repeatedly. Weekly remainder e-mails are sent once per week to participants. Participants are reminded that for stress management skills to improve they need to use the intervention as frequently as possible.

#### Waiting-list control

Participants in the waiting-list control group are asked to continue with their everyday life and come back for a secondary assessment after 30 days, after completing the secondary assessment they receive access to the intervention for one month.

### Outcomes

Self-report questionnaires are used for assessment of primary outcomes.

#### Primary outcomes

The main outcome measures are psychological well-being and severity of Adjustment disorder symptoms. Level of adjustment disorder symptom severity is evaluated with Adjustment disorder new model (ADNM-20) [28] questionnaire. This questionnaire is constructed of a stressors list and evaluation of symptoms. The list is composed of seven of acute events (e.g. divorce, moving) and nine chronic stressors (e.g. conflict with neighbours, serious illness). Participants are instructed to indicate all severe events from the list that they had experienced in the last 2 years and that caused significant level of stress. There is also a blank item for participants to fill in other significant stressors. Psychometric properties of the Lithuanian version of the ADNM-20 questionnaire are acceptable [7].

The WHO-5 Well-being Index (WHO-5) [29] is a short self-report measure for well- being assessment. Participants are asked to rate how each of the 5 statements applies to them when considering the last 2 weeks. Each item is scored from 5 (all of the time) to 0 (none of the time). The raw score therefore ranges from 0 (absence of well-being) to 25 (highest well-being). Participants who score > 16 on ADNM and < 50 on WHO-5 are included into the study.

#### Secondary outcomes

Secondary outcomes are motivation to use the program, expectations for the intervention and engagement into the program. These outcomes are measured with tools incorporated into the program and self-report questionnaires at assessment time points.

Motivation is measured by a three item questionnaire created by the authors of this study. Expectations are measured as the difference of two 10-point Likert scale questions: 1) *How do you feel today before starting the intervention BADI?* 2) *How do expect to feel after using the intervention BADI for one month?* Participants are also asked to indicate how often they are planning to use the intervention in the upcoming month and the reasons for choosing this intervention. Engagement is measured by the number of exercises completed by the participant.

### Analysis

#### Sample size

To achieve an improvement of 10 in mean scores of WHO-5 Well-being index we calculated about 73 participants per study arm to achieve 80 % power at α = .05. Factoring in the 30 % attrition rate, we seek to randomize a total of 190 participants.

#### Statistical analysis

All data will be analysed using Statistical Package for the Social Sciences (SPSS) version 20. Differences between groups at baseline will be investigated using *χ*^2^ tests of independence for categorical measures and t-tests for continuous measures. Intent-to-treat approach will be applied to the primary efficacy analysis from T1 to T2. Per-protocol and completer analysis will be performed as secondary. Mixed ANOVA with post hoc tests will be used to test effect of time and interactions between groups in time. Cohens’ *d* will be calculated for estimation of within and between group effect sizes.

Drop-out is considered if a participant uses the program at least once but does not complete self-report at T2. Engagement in this study is defined by the number of exercises completed by participant, i.e., the more exercises a participant completes the more engaged participant is considered.

### Discussion

Adjustment disorder might be a popular diagnosis [1], but there were only a few controlled clinical trials implemented to evaluate different treatment approaches for it [8]. First internet-based interventions for adjustment disorders were developed only recently and have not been evaluated in large scale empirical studies [8]. Therefore, we have developed BADI – a modular internet-based intervention for adjustment disorders. Users of this intervention do not have to complete all four modules (twelve exercises) in order to improve their condition. Rather they can test and choose modules and exercises which they like most and find useful. This is different from the linear approach, where users have to complete certain steps before they can progress to others.

We believe that a modular approach to internet-based intervention for adjustment disorders has several important benefits. Adjustment disorders can be caused by very different stressors therefore interventions should also reflect this variety, if they are targeting at the general population. Availability of different modules also means that users can choose according to their needs and preferences. And finally a modular approach provides flexibility for users – they can use the intervention whenever they need it and spend as much time as they feel appropriate. Thus it gives participants a sense of control, something that is important when facing stressful life events that may seem out of control.

BADI consists of 4 modules: Relaxation, Time management, Mindfulness and Strengthening relationships. It is based on stress and coping research and integrates evidence based modern treatment approaches such as Cognitive Behavioural therapy (CBT), mindfulness and body-mind practices, as well as exercises for enhancing social support. In this respect it differs from eHealth intervention developed by Maercker team [8], where PTSD treatment techniques such as written narrative exposition are also included.

In addition to evaluating effectiveness of BADI intervention, it will also examine correlations between effectiveness, motivation, expectations, drop-out and adherence. We believe that is important to look for new ways how to understand and measure drop-outs and adherence in the context of fully automated internet-based interventions as they are very different from traditional face-to-face interventions.

In case this RCT supports effectiveness of a fully automated version of BADI, it could be used very broadly. BADI could become a very cost-effective and accessible intervention for adjustment disorder.

## Abbreviations

BADI, brief adjustment disorder intervention; CBT, cognitive behavioural therapy; DSM, diagnostic and statistical manual of mental disorders; ICD, international classification of diseases; PTSD, post-traumatic stress disorder; RCT, randomized controlled trial.
